# Synthesis and Enzymatic Incorporation of Modified Deoxyuridine Triphosphates

**DOI:** 10.3390/molecules200813591

**Published:** 2015-07-24

**Authors:** Erkai Liu, Curtis H. Lam, David M. Perrin

**Affiliations:** Department of Chemistry, University of British Columbia, 2036 Main Mall, Vancouver, BC V6T1Z1, Canada; E-Mails: lekluck@chem.ubc.ca (E.L.); curtishonminglam@hotmail.com (C.H.L.)

**Keywords:** modified nucleoside triphosphates, Vent (exo-) DNA polymerase, incorporation

## Abstract

To expand the chemical functionality of DNAzymes and aptamers, several new modified deoxyuridine triphosphates have been synthesized. An important precursor that enables this aim is 5-aminomethyl dUTP, whereby the pendent amine serves as a handle for further synthetic functionalization. Five functional groups were conjugated to 5-aminomethyl dUTP. Incorporation assays were performed on several templates that demand 2–5 sequential incorporation events using several commercially available DNA polymerases. It was found that Vent (exo-) DNA polymerase efficiently incorporates all five modified dUTPs. In addition, all nucleoside triphosphates were capable of supporting a double-stranded exponential PCR amplification. Modified PCR amplicons were PCR amplified into unmodified DNA and sequenced to verify that genetic information was conserved through incorporation, amplification, and reamplification. Overall these modified dUTPs represent new candidate substrates for use in selections using modified nucleotide libraries.

## 1. Introduction

Systematic Evolution of Ligands by Exponential Enrichment (SELEX) was developed as a powerful methodology to select highly active nucleic acid ligands from an exceedingly large random library [1,2]. Over the past two decades, numerous aptamers have been selected to targets both large and small. Whereas SELEX has traditionally provided aptamers that bind ground states, similar combinatorial methods can be applied to select catalysts. As such, numerous DNAzymes have been selected for various reactions, most notably RNA cleavage [3,4,5], nucleopeptide bond formation [6], and DNA cleavage [7]. Compared to antibodies and enzymes, most aptamers and DNAzymes are functionality-poor, which may partially explain why, in general, nucleic acids have limited scope in terms of molecular recognition and catalysis [6,7,8]. While the major difference between aptamers and nucleic acid catalysts lies in the fact that the former bind ground states while the latter bind transition states, both will recognize targets with an ensemble of interactions that are characteristic of nucleic acids and not those seen in antibodies and catalysts. Nevertheless, the potential advantages of increasing the chemical diversity in one class of nucleic acid structure readily should apply to the other, despite some of the challenges involved in developing either aptamers or DNAyzmes as previously reviewed [9,10].

Starting over 15 years ago, we and many others have reported on the enzymatic incorporation of modified nucleoside triphosphates to produce modified DNA with additional functionalities. In many examples only one modified dXTP is incorporated [11,12,13,14,15,16,17,18,19,20,21,22,23,24,25,26,27,28,29,30,31,32,33,34], while in other cases two [35,36] and three modified nucleosides have been incorporated simultaneously in lieu of their unmodified counterparts [37,38,39,40]. In order to increase the chemical functionality of aptamers and DNAzymes, various nucleoside triphosphates must be first tested as substrates for various DNA polymerases. In addition, a DNA polymerase that can recopy modified DNA into unmodified DNA must be identified. In several cases, direct exponential amplification (PCR) of unmodified templates into detectable quantities of double-stranded modified DNA by high temperature polymerases has been shown [32,33]. While direct PCR of unmodified DNA into double stranded modified DNA is not essential for selection [35,36], the ability to perform PCR with modified dNTPs represents an added advantage as a single polymerase can be used for both steps that are required for selection and that the modified dNTP substrate is also capable of functioning during selection.

Despite the proliferation of reports on modified nucleosides that could be used for SELEX and related methods for catalyst selection, only a few publications have demonstrated successful combinatorial selection with modified dNTPs that results in a quantitative gain-of-function compared to unmodified aptamers and catalysts [41,42,43]. Nevertheless, key studies have demonstrated that functionalized nucleic acids indeed provide both quantitative and qualitative gains in function. For example, modified dUs were used to select against several protein targets against which high affinity unmodified aptamers could not be selected suggesting both quantitative and qualitative improvements in functional selection [31]. Similarly, several modified DNAzymes were selected using two or even three different modified nucleoside triphosphates for efficient M^2+^-independent RNA cleavage, a reaction that is notoriously difficult to catalyze with unmodified DNAzymes [22,35,36,38,39,44,45]. Taken together, these works highlight an enduring interest in generating new DNA-based polymers with added functionalities that are incorporated using polymerases and modified nucleoside triphosphates.

**Figure 1:**
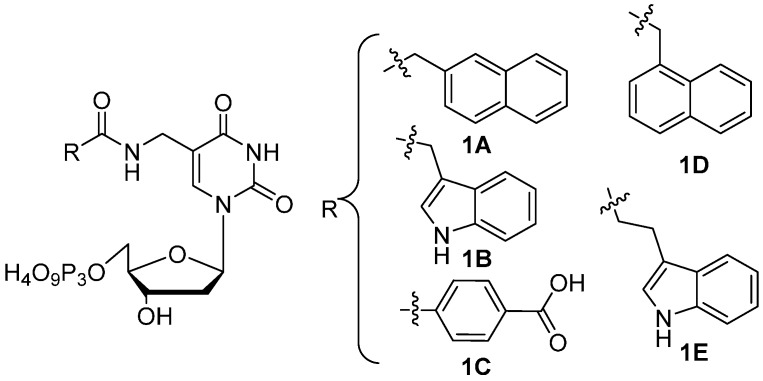
Modified dUTPs.

In an ongoing program in our lab to further expand the chemical functionality of *in vitro* selected DNA, here we report the synthesis of several modified nucleoside triphosphates (Figure 1). The design principle was based on a facile construction of 5-aminomethyl-dUTP that could be readily acylated with an NHS ester of a corresponding carboxylic acid. Compounds **1A** and **1D** represent hydrophobic/aromatic groups whereas compounds **1B** and **1E** are intended to mimic tryptophan. Compound **1C** contains a carboxylate group that would be expected to be a good chelator of various divalent metal ions such as Ca^2+^, Mg^2+^ and Zn^2+^. Following synthesis and purification, enzymatic incorporation of these modified nucleoside triphosphates were investigated with several polymerases on defined templates.

## 2. Results and Discussion

### 2.1 Primer Extension Assays 

Two templates were chosen to investigate incorporation: template **T1**, contains five consecutive adenines, which requires the polymerase to incorporate five modified dUTPs in a row, making this a very challenging sequence; template **T2** demands the incorporatation of eight modified nucleotide triphosphates in the full length product, yet there are at most only two consecutive modifications (Table 1). The primers and **T3** were employed to investigate an exponential PCR that ultimately uses templates containing modified nucleotides. **T3** contains eleven As in the template against which eleven modified dUTPs must be introduced to give a full length product. Therefore, **T3** is an equally challenging template compared to **T2**.

**Table 1:** Sequences of the primer and templates used in the primer extension assays. Red colored As indicate the position at which the polymerase must incorporate a modified dUTP.

	Sequences Used for Primer Extension or PCR Amplification
**P1**	5ʹ-TAATCGGGAAGGTCAGGGGGGAAAAGAAAA-3ʹ
**T1**	3ʹ-TTAGCCCTTCCAGTCCCCCCTTTTCTTTTAAAAAGTAACTAAGATGGACAGCTCC-5ʹ
**T2**	3ʹ-TTAGCCCTTCCAGTCCCCCCTTTTCTTTTGGCCAGTAACTAAGATCTACAGCTCC-5ʹ
**Primers**	5ʹ-GCGCTCGCGCGCCGCG-3ʹ 3ʹ-CGGGGAGGCGTCCGGCTGCG-5ʹ
**T3**	5ʹ-GCGCTCGCGCGCCGCGACGAACGAACGCCACACCAGACTACAGGCAGCCCCTCCGCAGGCCGACGC-3ʹ

The single nucleotide incorporation study was first conducted with the Sequenase v.2.0 DNA polymerase as it is one of the few DNA polymerases capable of incorporating 8-histaminyl-dA. All the five modified dUTPs were tested as substrates for Sequenase v.2.0 with both ddTTP and dTTP control lanes to provide defined products with several modified nucleotides including two instances where two modified dUs would be incorporated (**T2**) and one that would demand incorporation of five modified dUs in a row (**T1**).

**Figure 2:**
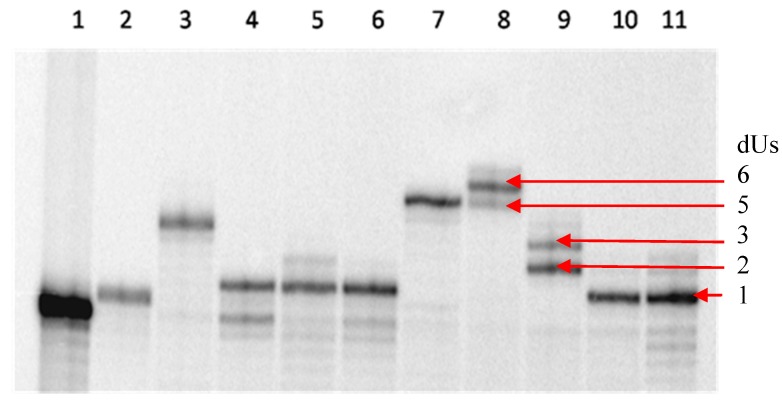
Screening various DNA polymerases with compound **1A** and **T1**. Lane 1, primer only; lane 2, ddTTP (control); lane 3, dTTP (control); lane 4, Tth; lane 5, Tfl; lane 6, Thermosequenase; lane 7, Vent(exo-); lane 8, Therminator; lane 9, KOD DASH; lane 10, Klenow; lane 11, Sequenase v.2.0.

In the single nucleotide incorporation experiment, all five modified nucleoside triphosphates were incorporated with primer and **T1** in the presence of Sequenase v.2.0 (Supplementary Information). The results indicated that Sequenase v.2.0 is only able to elongate one or two modified nucleotides, before stalling, irrespective of which modified dUTP was used. To improve the incorporation of the modified dUTPs, alternative polymerases were sought as a remedy. Ideally a polymerase would produce minimal truncation during incorporation. Because the worst incorporation was observed with the napthyl-modified dUTPs (in particular compound **1A**), a screening for polymerases using compound **1A** was commenced. The results are outlined in Figure 2, and indicate that, similar to Sequenase v.2.0 (lane 11 in Figure 2), most of the polymerases screened in this study only incorporated one modification (lanes 4–6 and 10). Most notably, Therminator DNA polymerase, known for its broad substrate scope, incorporated up to five modified nucleotides (lane 8) and even “overshot” its incorporation either due to its lower fidelity or more likely, the possibility of slippage at AT-rich regions. Similarly, Vent (exo-) DNA polymerase elongated five consecutive modified nucleotides (lane 7) while KOD DASH (lane 9) incorporated two to three modifications.

**Figure 3:**
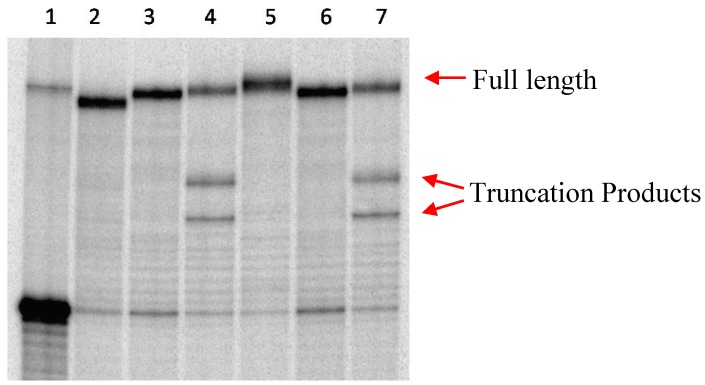
Full length incorporation with **T2** and Vent(exo-) DNA polymerase. Lane 1: primer only; lane 2: dATP, dGTP, dCTP + dTTP; lane 3: dATP, dGTP, dCTP + compound **1A**; lane 4: dATP, dGTP, dCTP + compound **1B**; lane 5: dATP, dGTP, dCTP + compound **1C**; lane 6: dATP, dGTP, dCTP + compound **1D**; lane 7: dATP, dGTP, dCTP + compound **1E**.

In light of these results, full length elongation with Vent(exo-) DNA polymerase was conducted. From Figure 2, it was known that Vent (exo-) DNA polymerase incorporates naphthyl compound **1A** well (Figure 2, lane 7). Unsurprisingly, full length product was observed for this incorporation (lane 3, Figure 3), with no truncation at all. Similar results were found for compounds **1C** and **1D** (lanes 5 and 6, Figure 3) wherein no truncation was found. Although compounds **1B** and **1E** produced full length product, some minor truncations were observed (lanes 4 and 7, Figure 3). In summary, Vent (exo-) DNA polymerase was found to be a good polymerase for the modified dUTPs synthesized in this project.

Following successful incorporation using Vent (exo-) DNA polymerase under primer extension conditions, an exponential PCR was attempted using unmodified templates. PCR experiments with modified nucleotides are much often more difficult than the primer extensions of the same substrates because a modified template must serve to direct the template incorporation of modified substrates. If the modified DNA strands are poor templates for polymerization, very little product will be produced.

**Figure 4:**
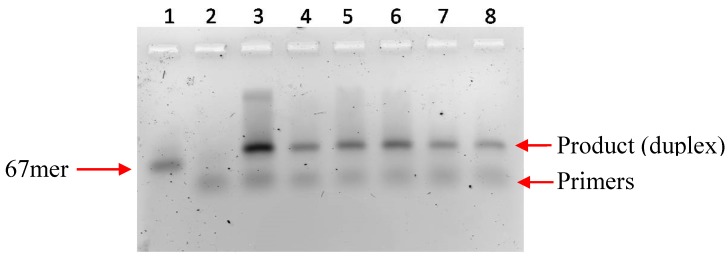
Agarose gel analysis of PCR products obtained with Vent (exo-). Total reaction mixture (20 μL) consisted of 1 pmole **T3**, 150 pmole of each primer, 0.2 mM of each dNTP and 2 unit of enzyme. Reactions were performed at 1X commercial thermopol buffer with a final Mg^+2^ concentration of 7 mM. Lane 1: 67mer DNA template; lane 2: no dNTP’s; lane 3: dATP, dGTP, dCTP + dTTP; lane 4: dATP, dGTP, dCTP + compound **1A**; lane 5: dATP, dGTP, dCTP + compound **1B**; lane 6: dATP, dGTP, dCTP + compound **1C**; lane 7: dATP, dGTP, dCTP + compound **1D**; lane 8: dATP, dGTP, dCTP + compound **1E**.

Figure 4 shows the PCR of a 67 nucleotide template (**T3**) using Vent (exo-) on an agarose gel. Compared to the lane 3 control, the modified product did not display any appreciable gel retardation on the agarose gel.

Although the PCR of all five modified dUTPs was not as good as that of natural dTTP, the results show generally satisfactory amplification in the presence of the modified dUTP. Based on the results in Figure 4, the modified DNAs serve as competent templates for the production of modified DNA.

To verify that each dUTP is correctly incorporated in keeping with Watson-Crick base-pairing rules, particularly over several cycles of PCR, 2% of the modified DNA from the PCR was isolated and reamplified into unmodified DNA to provide amplicons that were cloned, and sequenced. In each case, sequencing data matched that of the original template, which demonstrates that each dUTP can be incorporated faithfully and that the modified template can be recopied faithfully as well (see Supplementary Information).

The templated incorporation of modified dUTPs was initially investigated with Sequenase v.2.0 DNA polymerase. Since we had previously demonstrated that 5-(parahydroxybenzamidomethyl)-dUTP was a mediocre substrate for Sequenase v.2.0 [22], it was not unexpected that similar results would be obtained with these nucleosides. We attempted to optimize the incorporation using Sequenase v.2.0 in terms of temperature, substrate concentration, enzyme concentration and pH, yet no significant improvement was achieved (data not shown). While Sequenase-catalyzed incorporation was faltering, it is nonetheless possible to produce products of primer extension with several dUTPs incorporated. At the outset of this work, we chose Sequenase v.2.0 because it is one of the few DNA polymerases that can also accept the 8-histaminyl-dATP, a nucleoside that has provided several modified DNAzymes. Interestingly, Therminator and Vent (exo-) catalyzed good incorporation of all modified dUTPs, however of note, these high temperature polymerases are incapable of incorporating 8-histaminyl-dATP. Using these modified dUTPs, along with 8-histaminyl-dA may be possible with only with Sequenase v.2.0 yet not with either Therminator or Vent (exo-). Nevertheless if a selection that employs one modified dUTP is desired, Therminator or Vent (exo-) maybe the enzyme of choice.

A final note of caution is worthy of discussion: polymerases can be especially sensitive to both the modified nucleoside as well as nearest neighbor effects that can greatly affect the incorporation rate and efficiency. In the context of both 5-aminoallyl-dUTP and 8-histaminyl-dATP, we observed considerable variation in efficiency of incorporation of a modified dXTP based on the preceding sequence that may or may not contain modifications [35]. Despite generally poor incorporation efficiency, as well as considerable variability in terms of efficiency, both nucleosides were used successfully in a combinatorial selection [46]. When a different dUTP and dATP, which presented an imidazole and an amino group respectively, and which were incorporated with much greater efficiency [32,33], were used in selection, no greater catalytic activity was identified [44]. Hence, nucleoside triphosphates that are poorly incorporated may still give rise to robust catalytic activity [47]. By the same token, similarly modified nucleoside triphosphates that are more readily incorporated by PCR may result in catalytic activity that is less robust. While the trade-off between incorporation efficiency and selection outcome has been discussed at length elsewhere [48], to date, it is unclear how directly incorporation efficiency is correlated with robust selection outcomes.

## 3. Experimental Section

### 3.1. General Information

All starting materials were purchased from Sigma-Aldrich (St. Louis, MO, USA), Alfa Aesar (Ward Hill, MA, USA) and Fisher Scientific (Fisher Scientific, Pittsburgh, PA, USA), and used without further purification, unless noted. NMR spectra were recorded on a Bruker Avance 300 Spectrometer, ESI mass spectra were recorded on Bruker Esquire-LC, and HPLC was performed on an Agilent 1100 series instrument (Agilent, Santa Clara, CA, USA). UV spectra were recorded on a Beckmann DU 800 spectrophotometer. All incorporations were performed using autoclaved materials. Water was treated with diethyl pyrocarbonate (1 μL/10 mL) prior to autoclaving. Natural dNTPs and Klenow were purchased from Fermentas. 6X loading buffer, T4 polynucleotide kinase, Vent (exo–), Therminator and *Taq* DNA polymerase were purchased from New England Biolabs (Ipswich, MA, USA). Sequenase v.2.0 and Thermosequenase DNA polymerase were purchased from Affymetrix (Santa Clara, CA, USA). KOD-DASH DNA polymerase was purchased from EMD Millipore (Billerica, MA, USA). Tfl and Tth DNA polymerase were purchased from Promega (Madison, WI, USA). ^32^P-γ-ATP was purchased from Perkin Elmer (Waltham, MA, USA). Radioactivity was visualized using a Typhoon 9200 variable mode imager from GE Healthcare (Velizy-Villacoublay, France). All DNA oligonucleotides were purchased from Integrated DNA Technologies (Coralville, IA, USA).

### 3.2. Synthesis

**Scheme 1:**
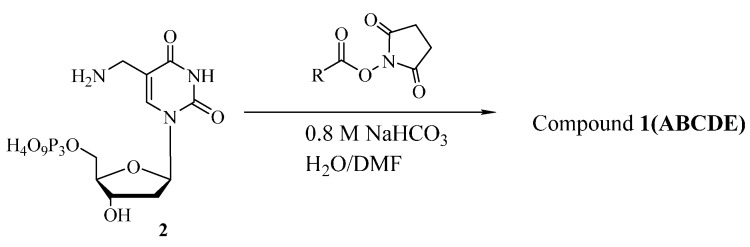
Synthesis of modified dUTPs.

The compound **2** was synthesized by following a previous report [22]. Then compound **2** was mixed with the NHS ester of five functional groups in the presence of sodium bicarbonate to give the desired products (Scheme 1).

Compound **1A**: 2-napthylacetic acid (0.382 g; 2 mmol), *N*-hydroxysuccinimide (0.253 g; 2.2 mmol) and EDC·HCl (0.421 g; 2.2 mmol) were added to a 25 mL RB flask, followed by the addition of DCM (15 mL). The reaction was stirred at room temperature overnight, after which TLC (EtOAc:hexane = 1:1) analysis indicated completion. Further DCM (15 mL) was added, and the reaction mixture was washed with saturated NaHCO_3_ (3 × 20 mL), 1M HCl (3 × 20 mL), dried over MgSO_4_, filtered, and evaporated to dryness *in vacuo*. TLC analysis showed only one spot, MS (ESI^+^): 306.6 (M + Na). The NHS ester (2.5 µmol) was added to DMF (5 µL), compound **2** (250 nmol) in H_2_O (10 µL), and 0.8 M NaHCO_3_ (10 µL). A mixture of the three solutions was stirred for 2 h, after which TLC analysis (dioxane:H_2_O:NH_3_·H_2_O = 6:4:1) indicated completion, The reaction mixture was purified on preparative TLC (dioxane:H_2_O:NH_3_·H_2_O = 6:4:1; Rf = 0.18). Then HPLC purification was carried out with a protocol described previously.[21] The synthesis and purification gave rise to 22 nmol (9%). Compound **1A** was quantified by the extinction coefficient of 21,000 cm^−^^1^·M^−^^1^ at 280 nm. MS (ESI^+^): *m*/*z* = 732.1 (M + 3Na − 2H), 754.0 (M + 4Na − 3H).

Compound **1B**: Compound **1B** was synthesized in the similar manner to compound **1A**. The synthesis and purification gave rise to 25 nmol (10% yield). Compound **1B** was quantified by the extinction coefficient of 14,300 cm^−^^1^·M^−^^1^ at 279 nm. MS (ESI^+^): *m*/*z* = 721.1 (M + 3Na − 2H), 743.0 (M + 4Na − 3H).

Compound **1C**: Compound **1C** was synthesized in the similar manner to compound **1A**. The synthesis and purification gave rise to 19 nmol (8% yield). Compound **1C** was quantified by the extinction coefficient of 17,500 cm^−1^·M^−1^ at 260 nm. MS (ESI^+^): *m*/*z* = 652.1 (M + Li), 674.1 (M + Na + Li).

Compound **1D**: Compound **1D** was synthesized in the similar manner to compound **1A**. The synthesis and purification gave rise to 28 nmol (11% yield). Compound **1D** was quantified by the extinction coefficient of 20,700 cm^−1^·M^−1^ at 280 nm. MS (ESI^−^): *m*/*z* = 663.9 (M − H), 685.9 (M + Na − 2H).

Compound **1E**: Compound **1E** was synthesized in a similar manner to Compound **1A**. The synthesis and purification gave rise to 25 nmol (10% yield). Compound **1E** was quantified by the extinction coefficient of 14,300 cm^−1^·M^−1^ at 279 nm. MS (ESI^−^): *m*/*z* = 667.1 (M − 1H), 689.1 (M + Na − 2H).

### 3.3. Enzymatic Incorporation

#### DNA Templates

(5ʹ to 3ʹ) **P1** TAATCGGGAAGGTCAGGGGGGAAAAGAAAA. **T1** CCTCGACAGGTAGAATCA ATGAAAAATTTTCTTTTCCCCCCTGACCTTCCCGATT. **T2** CCTCGACATCTAGAATCAATG ACCGGTTTTCTTTTCCCCCCTGACCTTCCCGATT. Two PCR primers GCGCTCGCGCGCCGCG and CGGGGAGGCGTCCGGCTGCG. **T3** GCCGCGACCACGCAACACCCACCTCGCCTACAAG CCCCTCCGCAGGCCGACGC

Primer extensions were conducted by following previous reports [21]. Reactions were prepared in a final incorporation volume of 10 μL reaction mixture consisting of 3 pmole 5ʹ-^32^P-labelled **Primer**, 3 pmole **T1**/**T2**. 0.02 unit pyrophosphatase, 50 µM of each dNTP and 1–6 units of DNA polymerase. Reaction mixture was incubated for 2 h at different temperature for different DNA polymerase. After the incubation, loading solution (20 μL) was added to each reaction and partial sample was loaded on denaturing PAGE (13%) and was visualized using a Phosphorimager (Molecular Dynamics–USA).

PCR reactions were conducted in a final volume of 20 μL reaction mixture consisted of 1 pmole **T3**, 150 pmole of each primer, 0.2 mM of each dNTP and 2 unit of enzyme. Reactions were performed at 1× thermopol buffer with a final Mg^2+^ concentration of 7 mM. The reaction was thermocycled for 30 cycles (15 s at 95 °C, 15 s at 58 °C and 40 s at 75 °C). A volume of 1 μL from each reaction was added to 4 μL water and 1 μL 6× DNA loading dye. Then the sample was loaded on 2% agarose gel containing 1% ethidium bromide and was visualized using a Phosphorimager.

### 3.4. Sequencing of the Modified DNA from PCR Amplification

All the five PCR reactions in the presence of the modified dUTPs were extracted with phenol/chloroform, 1/50 of the reaction was added to a new PCR reaction mixture consisting of 150 pmol of each primer (with overhanging 3ʹ-dAʹs), 0.2 mM of each dNTP and 2 unit of *Taq* DNA polymerase. The modified DNA as template was PCR amplified into natural DNA. The DNA was purified by 2% agarose gel electrophoresis. The collected DNA was inserted into pGEM-T-Easy Vector and standard TOPO cloning was performed with *E. coli* competent cells, and the cells were streaked onto lysogeny broth (LB) agar containing ampicillin (100 mg/L). Individual white colonies were picked and used to inoculate 1.5 mL SOC medium solution. The samples were cultured at 37 °C for 16 h. Plasmids were isolated by standard methods using a plasmid miniprep kit and bench-top centrifuge. The plasmid concentrations were quantified with a NanoDrop instrument and were restriction digested in the presence of EcoRI to screen for appropriately sized inserts. Afterwards, plasmids were submitted to the UBC NAPS Unit for sequencing.

## 4. Conclusions

In summary we have synthesized five modified nucleoside triphosphate analogues containing amino acid-like functional groups that may be important in aptamers and DNAzymes selections. We have explored this because the synthesis of the parent 5ʹ-amino-dUTP is especially robust and facile and thereby provides an accessible synthon for grafting various functionalities to give new dUTPs capable of functioning in a selection. These modified nucleoside triphosphates can be incorporated by Vent (exo-) DNA polymerase both in primer-extension reactions and PCR. This work demonstrates that all modified nucleoside triphosphates herein may find use in selections of modified aptamers and DNAzymes.

## Supplementary Materials

Supplementary materials can be accessed at: http://www.mdpi.com/1420-3049/20/08/13591/s1.
