# Supplementary material for: Synthesis and Enzymatic Incorporation of Modified Deoxyuridine Triphosphates
Source: Molecules. 2015 Jul 24;20(8):13591–602. doi: 10.3390/molecules200813591 (PMC6331837; doi:10.3390/molecules200813591)
Supplement: Supplementary file 1 [file molecules-20-13591-s001.pdf]

Supplementary Information

S1. Materials and Methods

All starting materials were purchased from Sigma-Aldrich, Alfa Aesar and Fisher Scientific, and used without further purification, unless noted. NMR spectra were recorded on a Bruker Avance 300 Spectrometer, ESI mass spectra were recorded on Bruker Esquire-LC, and HPLC was performed on an Agilent 1100 series instrument. UV spectra were recorded on a Beckmann DU 800 spectrophotometer.

HPLC purification was performed on an Agilent 1100 system using a Phenomenex Jupiter 10 μ C4 300 A column at a flow rate 1 mL/min. HPLC solvents contained two buffer systems: A, 50 mM triethylammonium acetate (pH 7.0) in ACN/water (1:1); B, 50 mM triethylammonium acetate (pH 7.0) in water. The purification was conducted with a step gradient as outlined in Table 1.

Table S1. HPLC solvent system.

Time (min)	0	10	18	19	24
% ACN/water	0	1	25	50	50

NMR, MS and UV Spectroscopy and HPLC purification

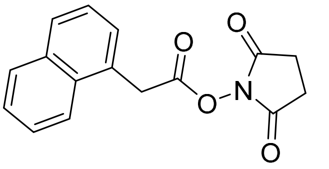

S1

Chemical Formula: $C_{16}H_{13}NO_4$
Molecular Weight: 283.28

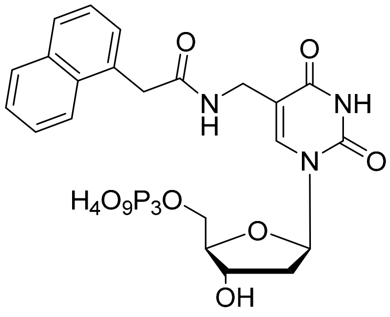

1A

Chemical Formula: $C_{22}H_{26}N_3O_{15}P_3$
Molecular Weight: 665.38

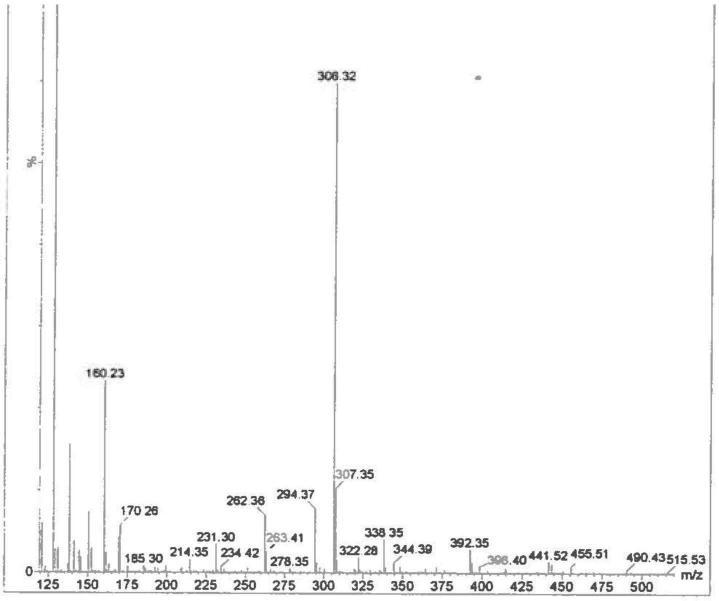

MS (ESI^+) for **S1**

Figure S1. *Cont.*

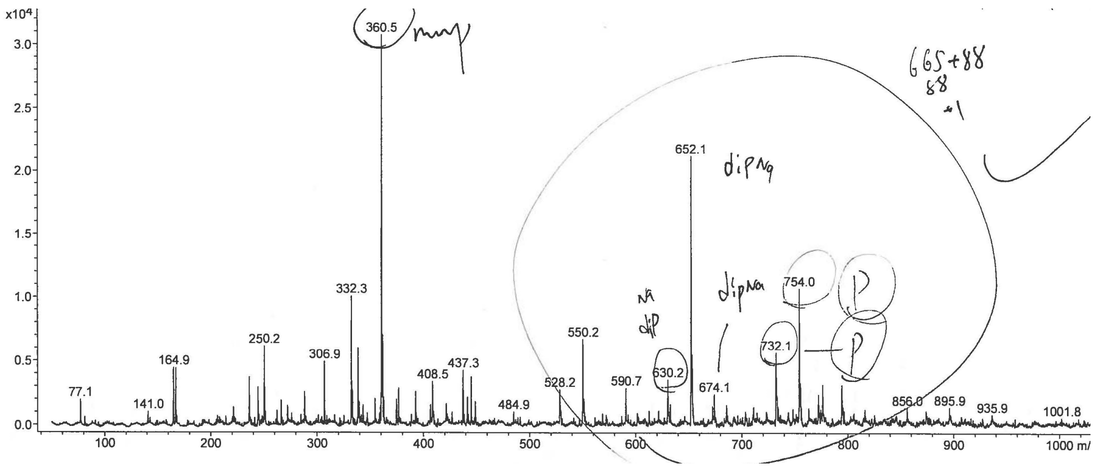

MS (ESI$^+$) for **1A**

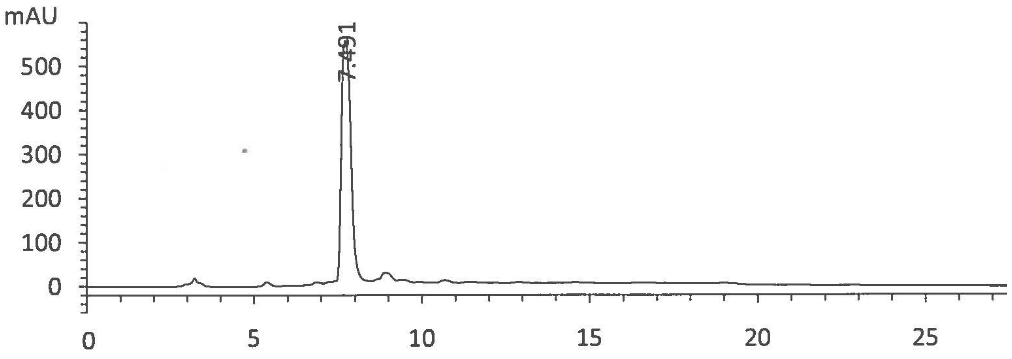

HPLC for **1A**

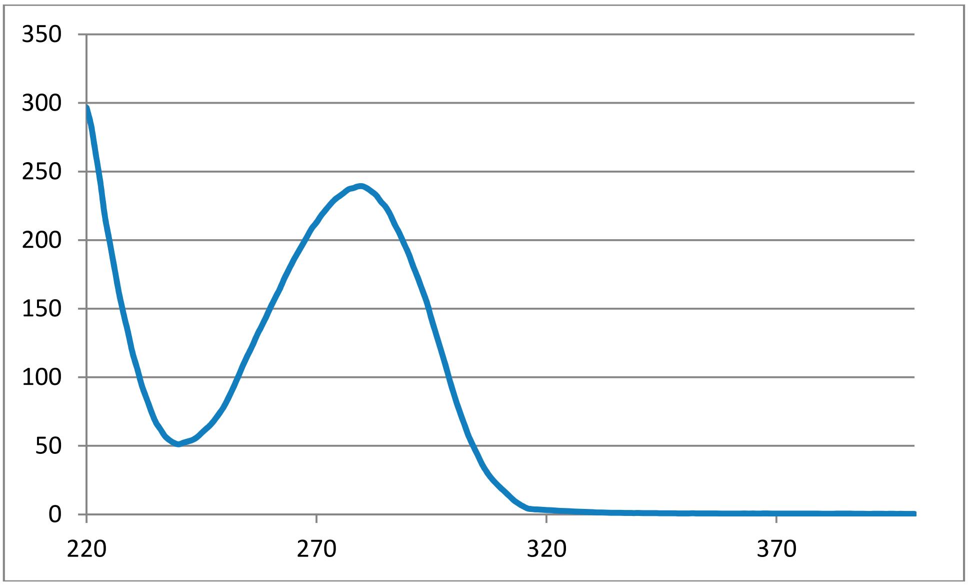

UV for **1A**

Figure S1. Compound **1A**: MS for **S1**, MS for compound **1A**, HPLC, UV.

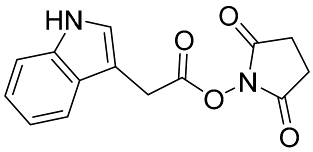

S2

Chemical Formula: $C_{14}H_{12}N_2O_4$
Molecular Weight: 272.26

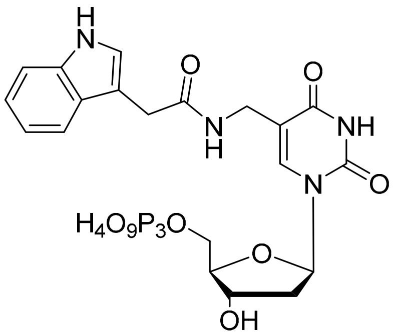

1B

Chemical Formula: $C_{20}H_{25}N_4O_{15}P_3$
Molecular Weight: 654.35

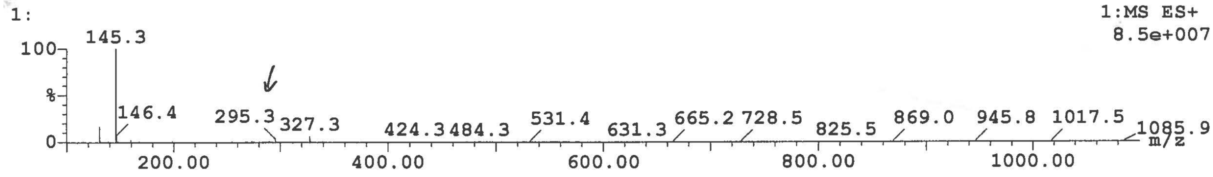

MS (ESI$^+$) for **S2**

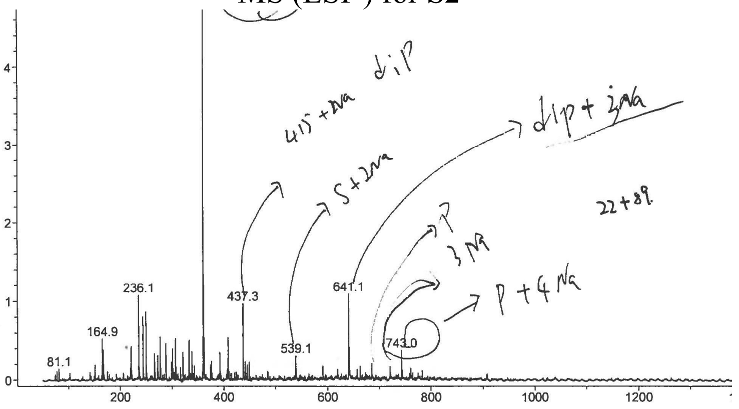

MS (ESI$^+$) for **1B**

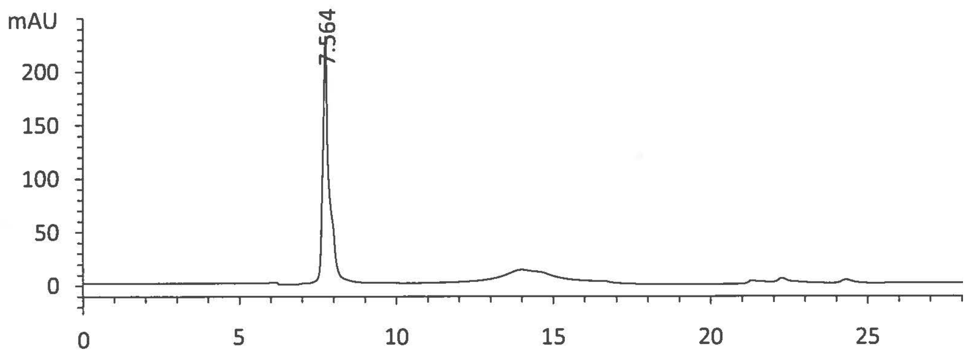

HPLC for **1B**

Figure S2. *Cont.*

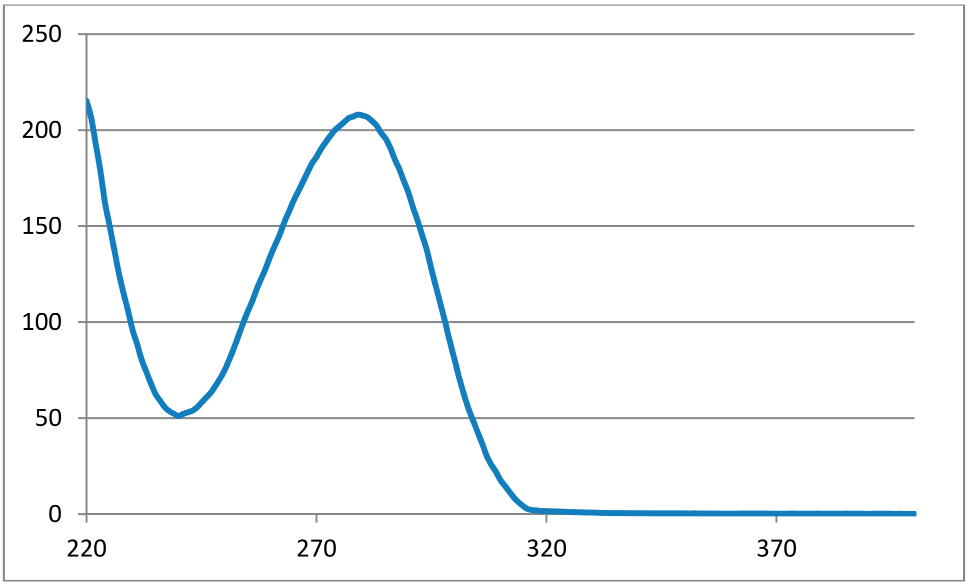

UV for **1B**

Figure S2. Compound **1B**: MS for modified group, MS, HPLC, UV for compound **1B**.

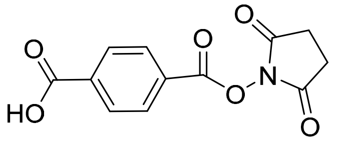

S3

Chemical Formula: $C_{12}H_9NO_6$
Molecular Weight: 263.21

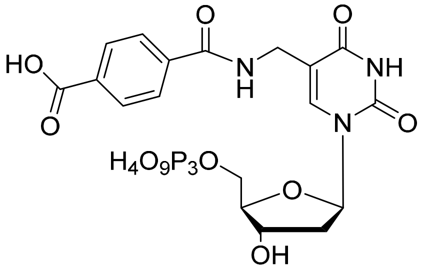

1C

Chemical Formula: $C_{18}H_{22}N_3O_{17}P_3$
Molecular Weight: 645.30

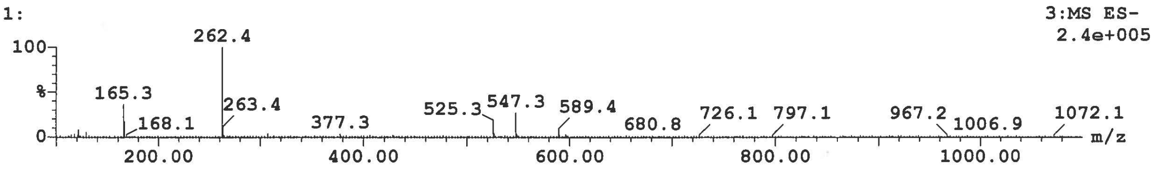

MS (ESI$^-$) for **S3**

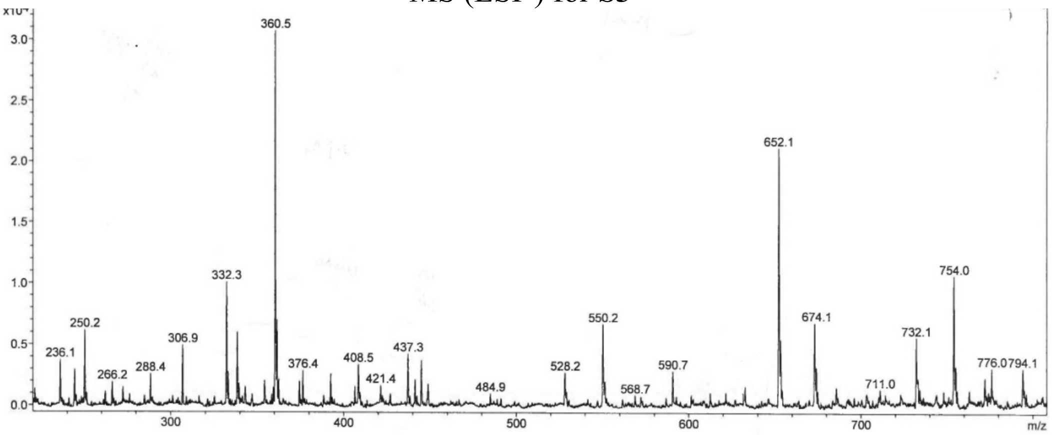

MS (ESI$^+$) for **1C**

Figure S3. *Cont.*

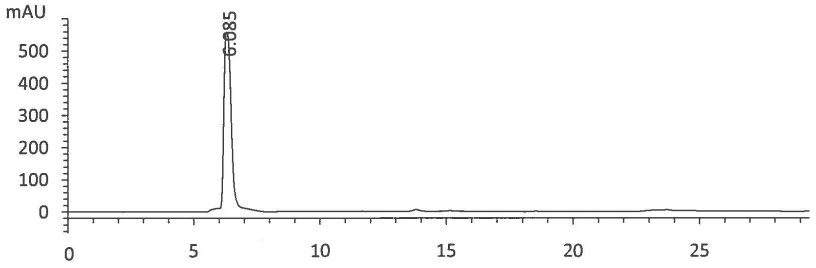

HPLC for **1C**

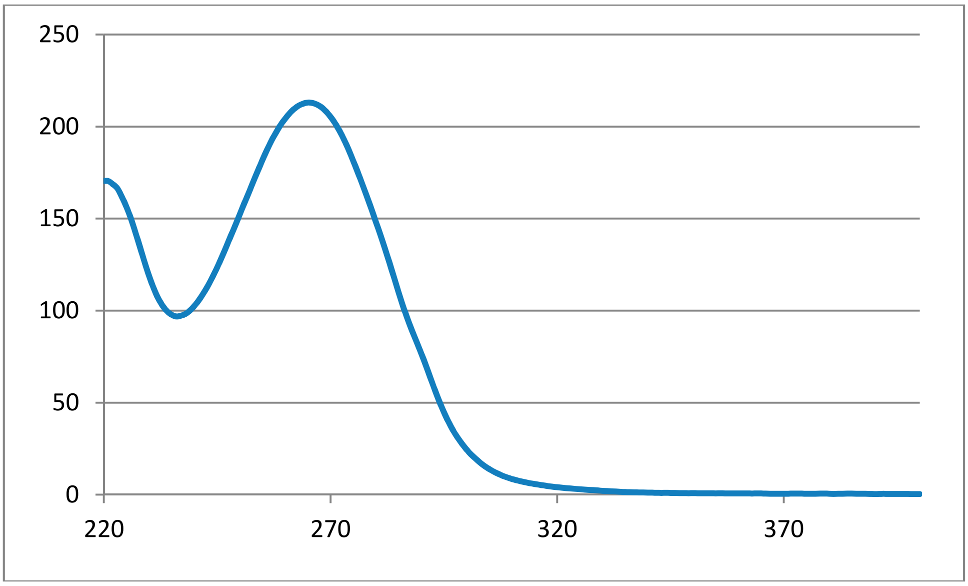

UV for **1C**

Figure S3. Compound **1C**: MS for modified group, MS, HPLC, UV for compound **1C**.

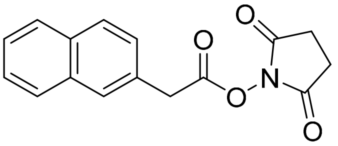

S4

Chemical Formula: $C_{16}H_{13}NO_4$
Molecular Weight: 283.28

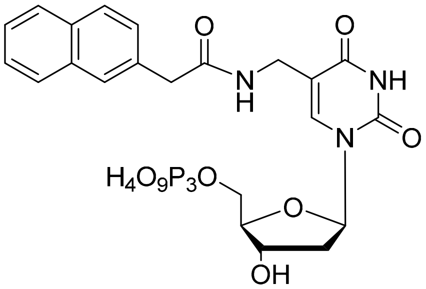

1D

Chemical Formula: $C_{22}H_{26}N_3O_{15}P_3$
Molecular Weight: 665.38

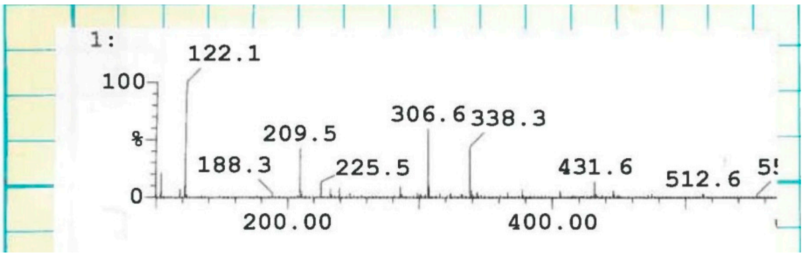

MS (ESI$^+$) for **S4**

Figure S4. *Cont.*

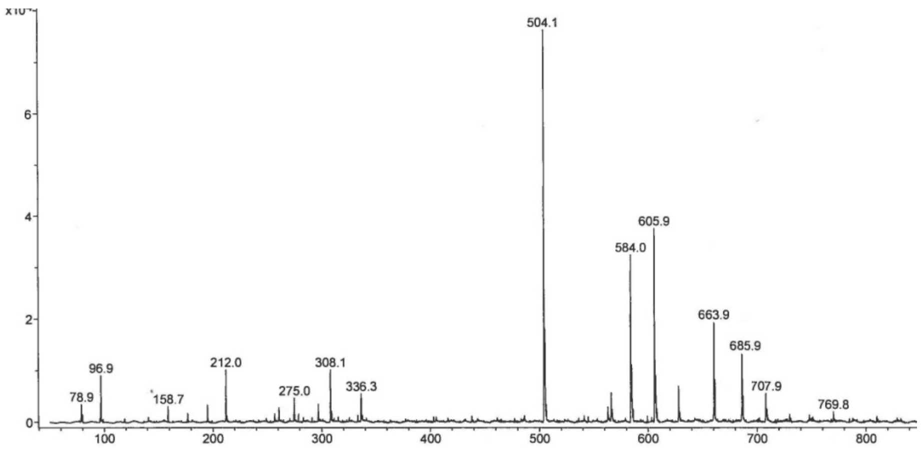

MS (ESI$^-$)for **1D**

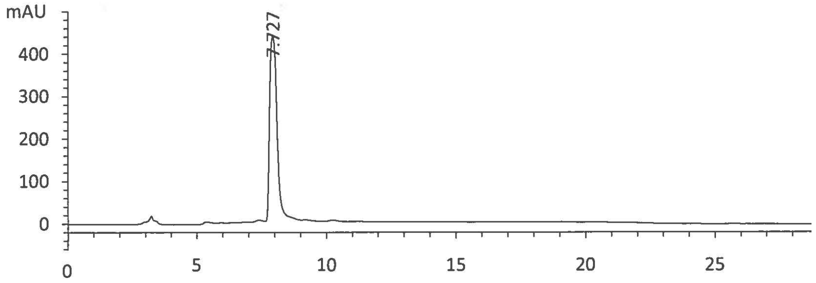

HPLC for **1D**

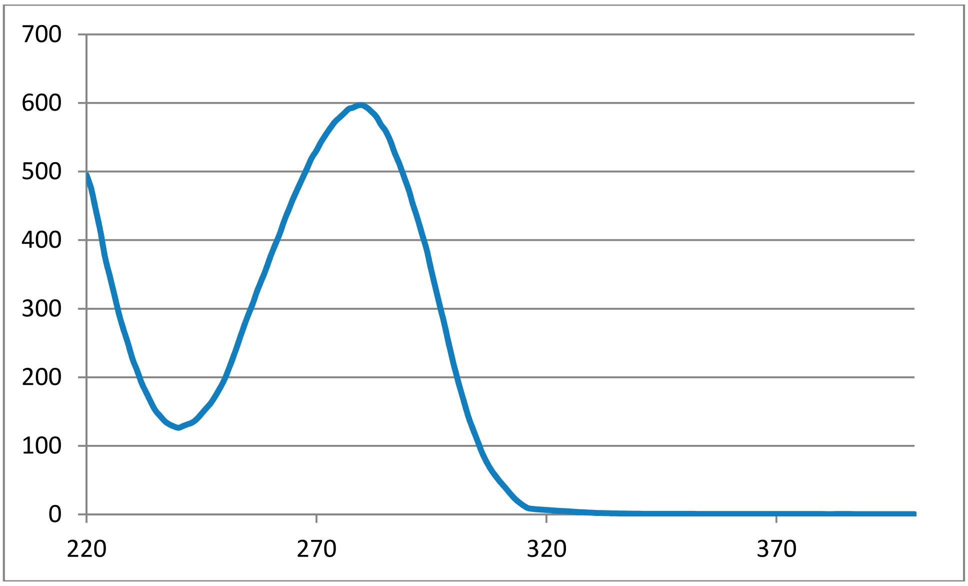

UV for **1D**

Figure S4. Compound **1D**: MS for modified group, MS, HPLC, UV for compound **1D**.

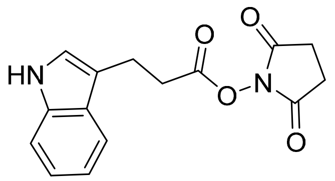

S5

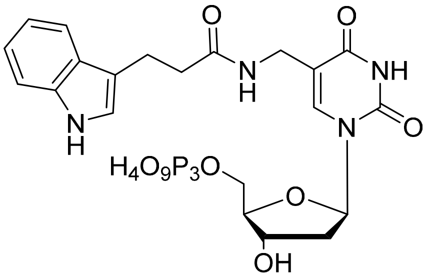

1E

Chemical Formula: $C_{15}H_{14}N_2O_4$
Molecular Weight: 286.29

Chemical Formula: $C_{21}H_{27}N_4O_{15}P_3$
Molecular Weight: 668.38

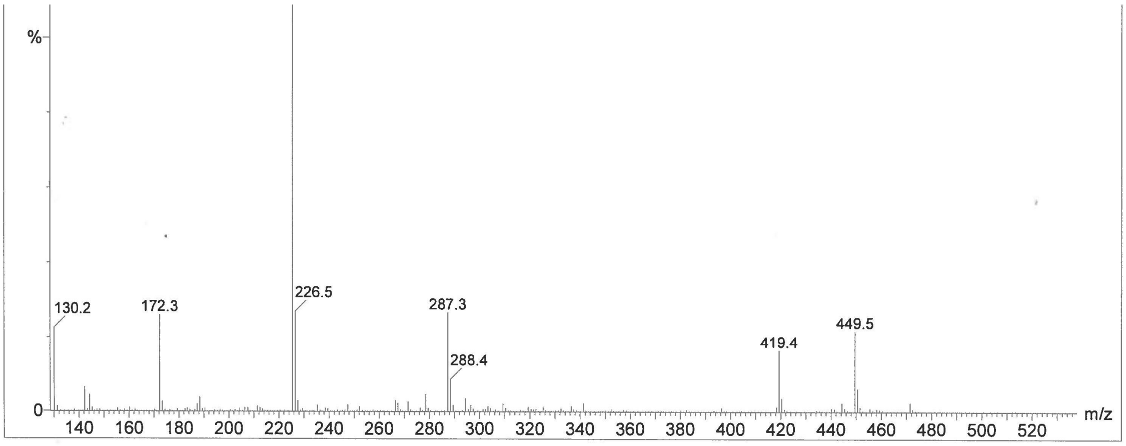

MS (ESI$^+$) for **S5**

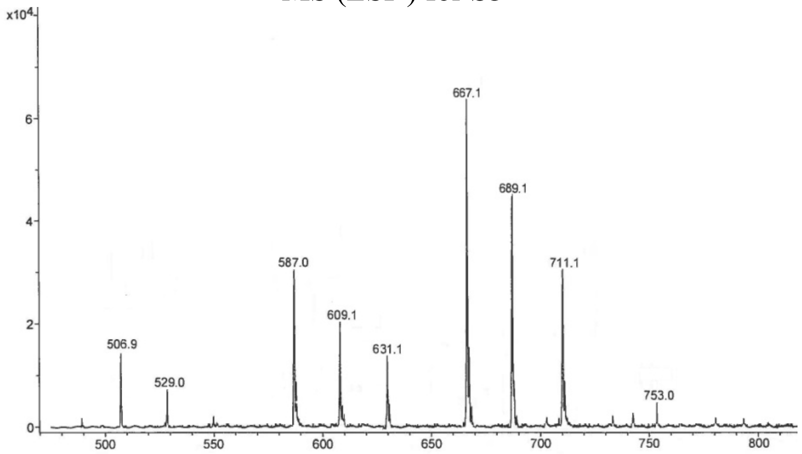

MS (ESI$^-$) for **1E**

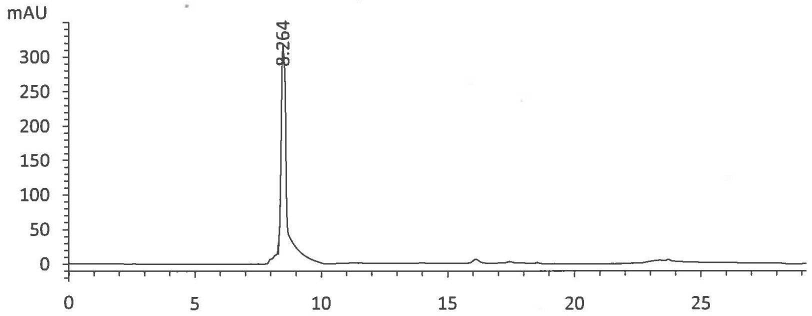

HPLC for **1E**

Figure S5. *Cont.*

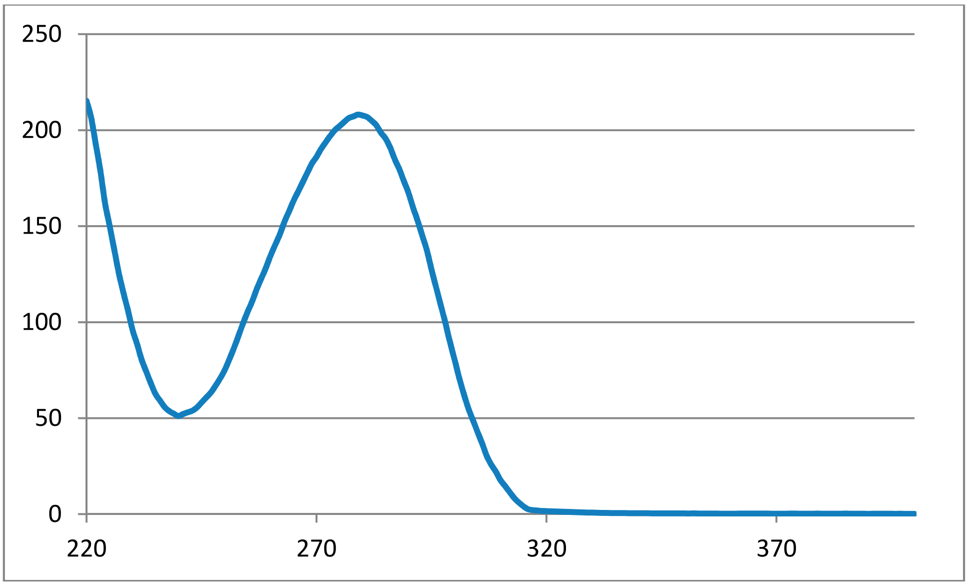

UV for **1E**

Figure S5. Compound **1E**: MS for modified group, MS, HPLC, UV for compound **1E**.

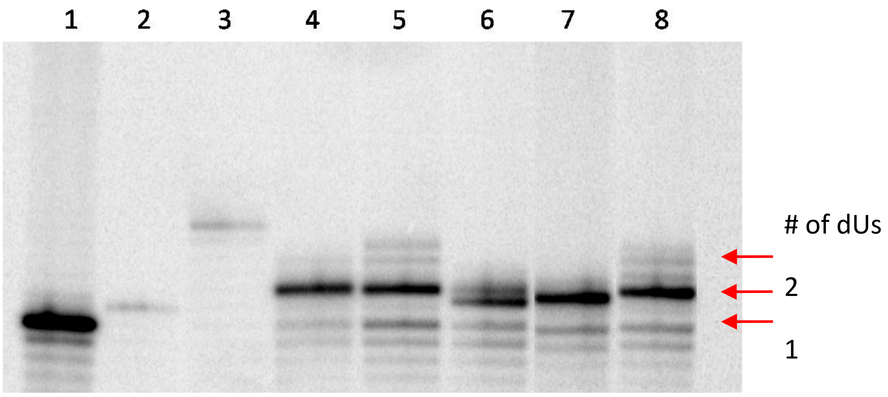

Figure S6. Single nucleoside triphosphate incorporation test (**T1**) with Sequenase. Lane 1, primer; lane 2, ddTTP (control; lane 3, dTTP (control); lane 4, compound **1A**; lane 5, compound **1B**; lane 6, compound **1C**; lane 7, compound **1D**; lane 8, compound **1E**

Sequencing Results

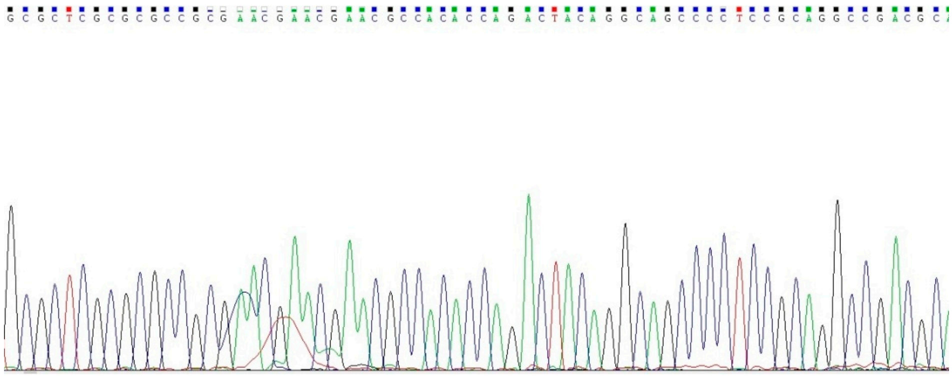

Figure S7. The sequencing data included two primer regions for the dTTP PCR amplicon (control—no modifications).

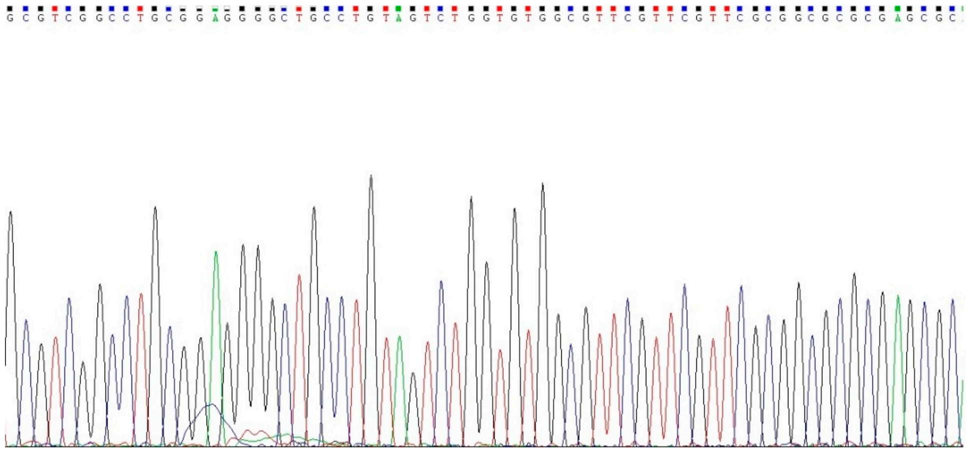

Figure S8. The sequencing data included two primer regions for the compound **1A** PCR amplicon.

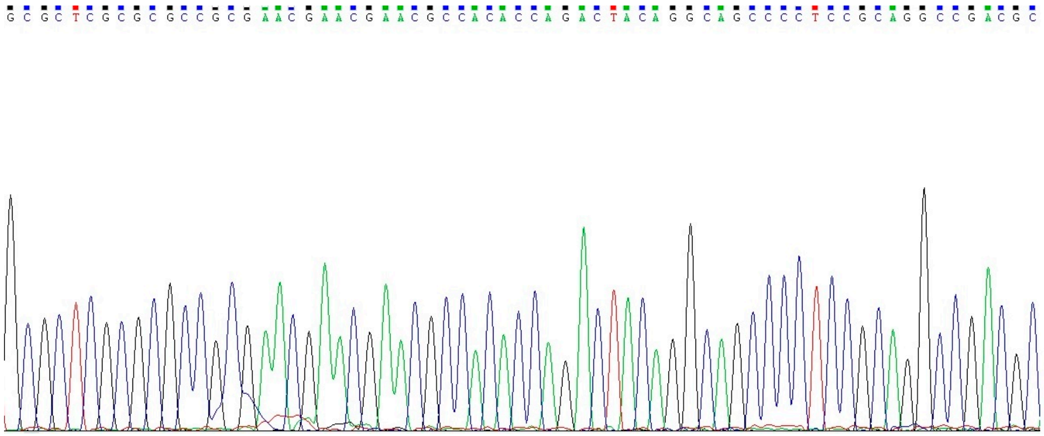

Figure S9. The sequencing data included two primer regions for the compound **1B** PCR amplicon.

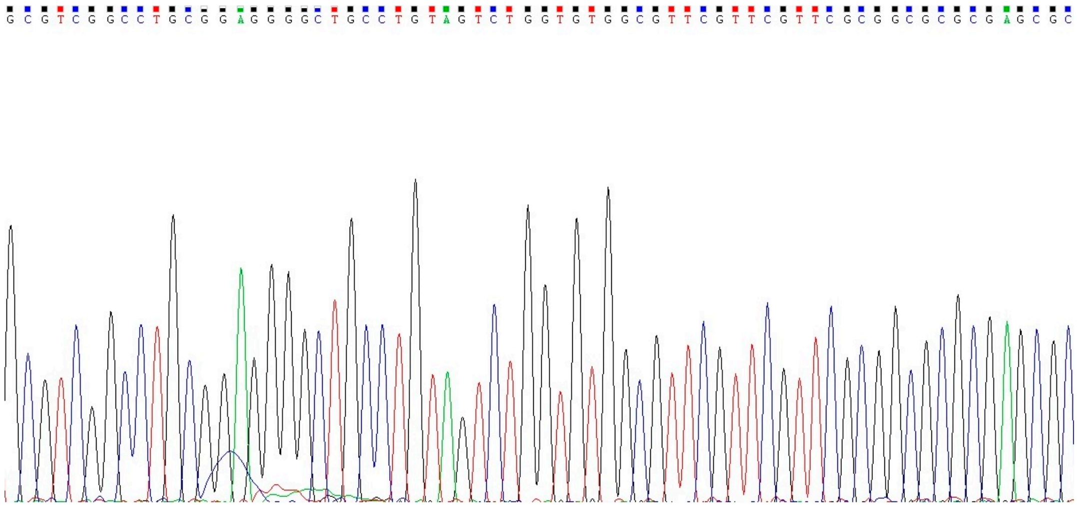

Figure S10. The sequencing data included two primer regions for the compound **1C** PCR amplicon.

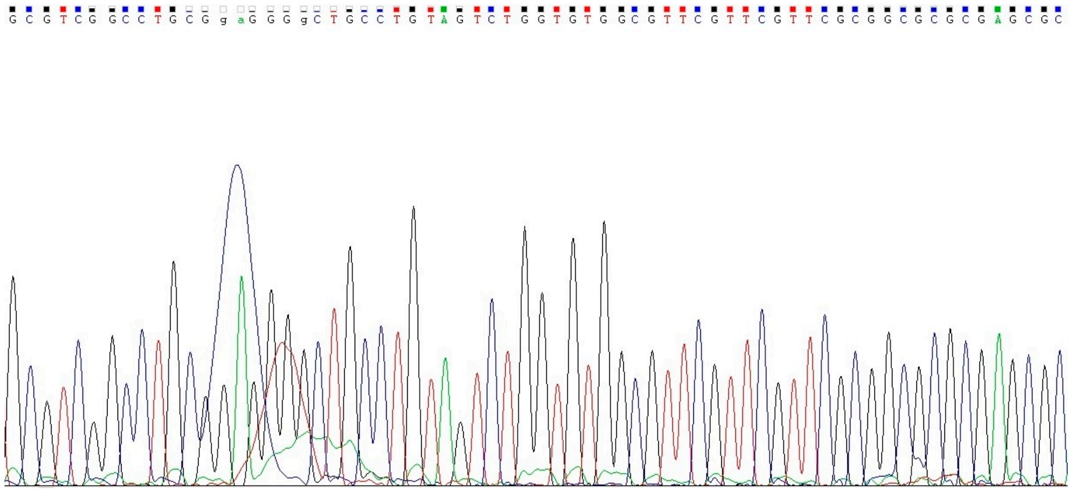

Figure S11. The sequencing data included two primer regions for the compound **1D** PCR amplicon.

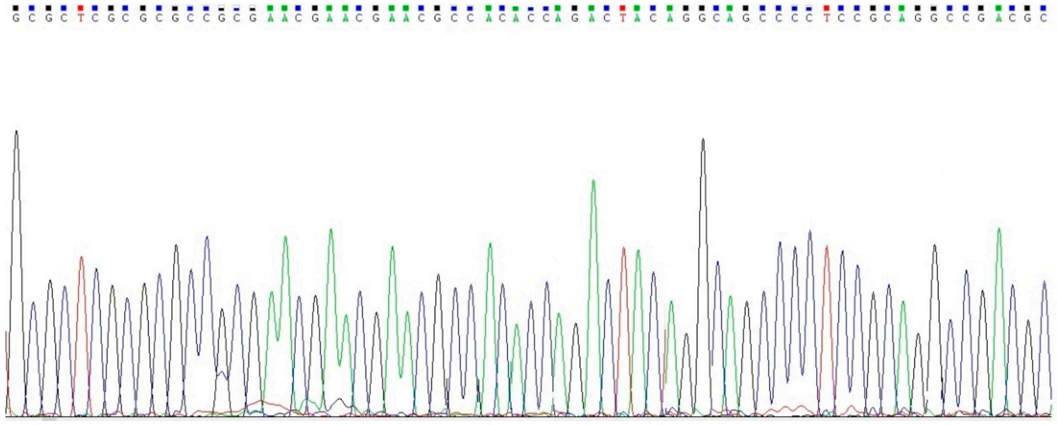

Figure S12. The sequencing data included two primer regions for the compound **1E** PCR amplicon.
